# Double-diabetes in a real-world sample of 2711 individuals: associated with insulin treatment or part of the heterogeneity of type 1 diabetes?

**DOI:** 10.1186/s13098-016-0143-7

**Published:** 2016-03-22

**Authors:** Fernando M. A. Giuffrida, Caroline Bulcão, Roberta A. Cobas, Carlos Antonio Negrato, Marilia B. Gomes, Sergio Atala Dib, on behalf of the Brazilian Type 1 Diabetes Study Group (BrazDiab1SG)

**Affiliations:** Departamento de Ciências da Vida, Universidade do Estado da Bahia (UNEB), Rua Silveira Martins, 2555, Cabula, Salvador, Bahia CEP: 41.150-000 Brazil; Centro de Diabetes e Endocrinologia do Estado da Bahia (CEDEBA), Salvador, Brazil; Universidade Estadual do Rio de Janeiro, Rio De Janeiro, Brazil; Associação de Diabéticos de Bauru, Bauru, Brazil; Centro de Diabetes, Universidade Federal de São Paulo, São Paulo, Brazil

**Keywords:** Double diabetes, Type 1 diabetes, Cardiovascular risk factors

## Abstract

**Background:**

Double diabetes (DD) describes both individuals with obesity upon diagnosis of type 1 diabetes and those who have gained weight during follow-up, although cardiovascular risk factors (CVRF) are not well understood in this group. We aim to evaluate the frequency of DD in a real-world type 1 diabetes sample and the interaction of insulin treatment with CVRF.

**Methods:**

Multicentre cross-sectional study of 2711 individuals with clinical diagnosis of type 1 diabetes from secondary diabetes centres in 20 Brazilian cities.

**Results:**

Patients with diabetes duration <5 and ≥5 years had similar frequency of overweight (20.4 vs. 25 %) and obesity, (9.8 vs. 6.1 %), p 0.28 for trend. Insulin dose (U/kg/day) was lower in obese individuals compared to normal BMI, with mean (95 % CI) 0.72 (0.62–0.83) vs. 0.88 (0.84–0.92) U/kg/day for diabetes duration <5 years and 0.84 (0.77–0.92) vs. 0.99 (0.97–1.01) U/kg/day for duration ≥5 years. Obese individuals had lower HDL (47.5 vs. 54.4 mg/dL) and higher non-HDL-cholesterol (134.5 vs. 115.2 mg/dL) than lean ones only among those with more than 5 years of diabetes.

**Conclusions:**

Lower insulin doses in obese individuals point to a role of clinical heterogeneity in insulin deficiency rather than normal progression of type 1 diabetes. Early obesity in type 1 diabetes is associated to lower HDL-cholesterol and higher number of CVRF. These data suggest a broad landscape of pathophysiological phenomena in double diabetes, rather than simple progression of a homogeneous clinical entity.

**Electronic supplementary material:**

The online version of this article (doi:10.1186/s13098-016-0143-7) contains supplementary material, which is available to authorized users.

## Background

Type 1 diabetes is caused by autoimmune destruction of beta-cells, leading to absolute insulin deficiency during its natural history. For this reason, type 1 diabetes has been associated, until almost a decade ago, with a leaner phenotype and absence of other cardiovascular risk factors upon diagnosis [1]. However, obesity is often recognised in individuals with type 1 diabetes already at diagnosis, owing partly to its rising incidence in the general population. Not only is obesity compatible with a type 1 diabetes diagnosis, but it is also potentially a risk factor for its development [2].

Double-diabetes (DD) was a term coined to describe individuals with type 1 diabetes showing clinical features compatible with type 2 diabetes [3]. It has been variably used in literature, to describe both individuals with obesity and other insulin resistance (IR) characteristics since diagnosis and those who have gained weight during follow-up, becoming obese over time [4]. Definition of a strict intermediate subtype between both types of diabetes is difficult, therefore this grey zone between them behaves more like a continuum according to current evidence [1].

Insulin treatment mimics endogenous insulin secretion imperfectly, thereby exposing individuals with type 1 diabetes to a hyperinsulinaemic environment. This can contribute to weight gain and development of clinical features associated with IR. Insulin dose, in this case, could be regarded as a surrogate marker of this hyperinsulinaemic environment, analogously to plasma insulin in non-diabetic individuals with the metabolic syndrome [5].

However, the relationship between this hyperinsulinaemic environment and metabolic factors in a milieu without primary IR is still in discussion. The various DCCT/EDIC studies constitute an invaluable source of knowledge about the progression of obesity and cardiovascular risk factors in type 1 diabetes. Nevertheless, by the time the DCCT began recruitment, BMI was considered a tool for the clinical distinction between type 1 and type 2 diabetes, leading obese patients (i.e., those above 130 % of ideal body weight) to be excluded from the trial [6]. Therefore, this sample is possibly not representative of real-life type 1 diabetes today [7]. In UKPDS, likewise, individuals were considered to have type 2 diabetes solely based on age, milder hyperglycaemia, and absence of ketonuria [8]. However, distinction between type 1 and type 2 diabetes is not always straight forward. Up to 15 % of newly diagnosed individuals cannot have a diabetes type properly assigned [9, 10]. Therefore, differences between these two major subtypes of diabetes are becoming hazy [1], leading even to proposals of declassifying the disease [11].

Cardiovascular risk factors such as dyslipidaemia and hypertension can be associated with type 1 diabetes. The interaction of intensive insulin treatment with them is not well understood, since intensive therapy can at the same time diminish cardiovascular risk (through better metabolic control) and worsen these factors via weight gain [2]. Thus, this complex interplay must be further elucidated. Adequate understanding of these mechanisms could possibly lead to better therapeutic approaches.

This study aims to: (1) verify the frequency of overweight and obesity in a real-world sample of Brazilian individuals with type 1 diabetes; (2) evaluate the association of insulin treatment with IR traits and cardiovascular risk factors.

## Methods

**Fig. 1:**
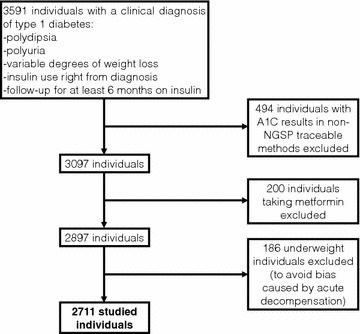
Flowchart of initial sample, excluded patients and final number of studied individuals

An initial sample of 3591 individuals with type 1 diabetes from the Brazilian Type 1 Diabetes Study Group was studied cross-sectionally. Patients have been recruited in 28 secondary and tertiary diabetes centres, located in 20 cities in all five major geographic regions of Brazil. Recruitment period was from December 2008 to 2012. Inclusion criteria were a clinical diagnosis of type 1 diabetes, i.e., typical clinical symptoms (polydipsia, polyuria and varying degrees of weight loss), insulin therapy requirement since diagnosis without interruption, and being followed at the diabetes center by at least 6 months prior to recruitment. Data were collected from medical records by a standardised medical chart review form [12]. After exclusion of 880 individuals by various criteria (described on the flowchart depicted in Fig. 1), a final sample of 2711 individuals was studied.

The following variables have been recorded: gender, age at recruitment, diabetes duration, total insulin dose, insulin dose per body weight, bolus/total insulin dose ratio, number of insulin applications (the sum of all basal and prandial insulin applications, used to characterise insulin regimen), BMI, body surface area (calculated by the DuBois equation), systolic blood pressure (SBP), diastolic blood pressure (DBP). Mean blood pressure (MBP) was calculated as [SBP + 2 × DBP]/3. Fasting plasma glucose (FPG), HbA1c, total cholesterol, HDL-cholesterol, and triglycerides were dosed by previously described methods at each center (12). LDL-cholesterol was calculated by Friedwald’s equation. Non-HDL-cholesterol was calculated as total cholesterol minus HDL. Familial history of type 2 diabetes in first-degree relatives, presence of overt nephropathy (defined as AER >30 mg/24 h, renal failure, dialysis treatment, or renal transplantation), coronary events (myocardial infarction, coronary bypass, or transluminal angioplasty) and number of cardiovascular risk factors (low HDL, high LDL, hypertriglyceridaemia, arterial hypertension) were also studied. Low HDL was defined as HDL-cholesterol below 50 mg/dL in women and 40 mg/dL in men. High LDL was defined as LDL-cholesterol above 100 mg/dL. Hypertriglyceridaemia was defined as triglycerides above 150 mg/dL. Arterial hypertension was defined as blood pressure above 140 × 90 mmHg in adults and above the 95th percentile for age and stature in children and adolescents or medical record reporting the diagnosis. Use of antihypertensive medication was not used as criterion in order not to overestimate the number of patients with hypertension by including normotensive individuals using medication only for renal protection.

Individuals have been divided into three groups according to BMI status: normal (BMI < 25 kg/m^2^ in adults or below the 85th percentile for those younger than 18); overweight (BMI between 25 and 29.9 kg/m^2^ for age ≥18 or between percentiles 85 and 94.9 for age <18); obesity (BMI ≥ 30 kg/m^2^ or percentile ≥95). Patients were also divided in two groups according to diabetes duration: ≥5 years and <5 years. There is evidence of significant weight gain in type 1 diabetes after approximately 5 years of diabetes duration in literature. This cut point also minimises the impact of recovering weight lost during acute decompensation [13, 14]. Moreover, five years can be regarded as an upper time limit for significant residual beta-cell function [15].

Frequencies of normal weight, overweight, and obese individuals have been compared between both diabetes duration subgroups by Mantel–Haenszel test and p value for linear-by-linear association was calculated.

After splitting the sample in six groups according to BMI status and diabetes duration, continuous variables have been transformed into square root, log, or inverse according to skewness of distribution. Insulin dose per body weight, total number of insulin applications, HbA1c, HDL-cholesterol, non-HDL-cholesterol, and MBP were separately entered as dependent variables in factorial ANCOVA models. The two lipid variables and MBP were chosen in order to illustrate the cardiovascular risk factor profile while avoiding multicollinearity among correlated variables. BMI status, diabetes duration, familial history of type 2 diabetes, and gender have been entered as fixed factors. Continuous variables above have been entered as covariates for each other. Age and body surface area have also been entered as covariates, to minimise possible bias caused by the difference of age between diabetes duration groups, as well as in the interpretation of insulin doses per body weight.

Missing data have been excluded list-wise. Post-hoc power analyses have been performed in the ANCOVA models. Type IV sum of squares was used for significance testing due to unequal group size. Partial eta-squared (η^2^), denoting percentage of total variance in the dependent variable accounted for by the independent variable, p values, and power were recorded. Only findings with power above 80 % have been reported. An alpha level of 5 % was considered significant.

Statistical analyses have ben conducted using SPSS 13.0 Statistical Package (SPSS Inc., Chicago, IL). The study has been previously approved by each centre’s Research Ethics Committee, as previously described [12].

## Results

**Fig. 2:**
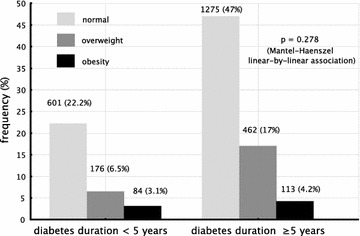
Relative frequencies of normal, overweight, and obese individuals, according to diabetes duration

Clinical and laboratory features of individuals are depicted in Table 1. Frequencies of normal BMI, overweight, and obesity were similar in individuals with diabetes duration below and above or equal to 5 years. In individuals with less than 5 years of diabetes, 69.8 % had normal BMI, 20.4 % were overweight, and 9.8 % were obese. In those with 5 or more years of diabetes duration, 68.9 % had normal BMI, 25 % were overweight, and 6.1 % were obese. Linear-by-linear association between BMI status and diabetes duration was not significant, with p = 0.278 (Fig. 2).

**Table 1:** Clinical and laboratory features of studied individuals according to diabetes duration and BMI status (univariate analysis)

	Diabetes duration <5 years			p	Diabetes duration ≥5 years			p
	Normal	Overweight	Obesity		Normal	Overweight	Obesity	
n	601	176	84		1275	462	113	
Female gender (%)	54.4	56.3	50	NS	55.5	59.3	67.3	0.01*
Age (years)	13.5 (6.6)	13.1 (8.0)	9.8 (6.6)	<0.001	24.2 (11.5)	26.3 (12)	25.6 (13.9)	0.003
Diabetes duration (years)	2.32 (1.27)	2.20 (1.27)	2.20 (1.30)	NS	12.9 (7.4)	14.1 (8)	13.9 (9.3)	NS
Total insulin dose (U)	39.1 (21.8)	38.3 (21.0)	36.6 (23.7)	NS	51.7 (21.3)	59.1 (21.2)	60.1 (25.1)	<0.001
Insulin dose per body weight (U/kg)	0.90 (0.42)	0.82 (0.32)	0.83 (0.32)	NS	0.95 (0.38)	0.88 (0.34)	0.81 (0.34)	<0.001
Bolus/total insulin dose ratio (%)	22.6 (14.8)	21 (14.6)	20.8 (13)	NS	21.8 (14.7)	23.4 (15.1)	24.3 (15.1)	0.049
Number of insulin doses	4.71 (1.19)	4.66 (1.27)	4.90 (1.23)	NS	4.85 (1.20)	5.0 (1.20)	5.36 (1.17)	<0.001
BMI (kg/m^2^)	18.7 (2.66)	21.4 (3.55)	23.4 (5.21)	<0.001	21 (2.36)	25.5 (2.49)	29.5 (4.58)	<0.001
Body surface area (m^2^)	1.33 (0.35)	1.36 (0.40)	1.23 (0.45)	0.03	1.57 (0.24)	1.72 (0.23)	1.77 (0.30)	<0.001
Systolic blood pressure (mmHg)	101.6 (13.8)	102.7 (14.5)	103.1 (16.5)	NS	113.1 (16.3)	117.7 (17.4)	117.6 (16.7)	<0.001
Diastolic blood pressure (mmHg)	65.7 (10.4)	66.8 (11.7)	66.6 (9.7)	NS	72.3 (11)	74.4 (11.1)	74.2 (11.7)	0.001
Fasting plasma glucose (mmol/L)^a^	9.6 (5.8)	9.6 (5.4)	9.8 (4.9)	NS	10.1 (5.8)	10.2 (5.7)	9.9 (6.1)	NS
HbA1c (%)	9.2 (2.5)	8.9 (2.2)	8.8 (2.4)	NS	9.3 (2.3)	9.3 (2.3)	9.3 (2.3)	NS
HbA1c (mmol/mol)	78 (27)	74 (25)	73 (26)	NS	78 (25)	78 (25)	78 (25)	NS
Total cholesterol (mmol/L)^b^	4.20 (1.02)	4.18 (0.81)	4.27 (0.87)	NS	4.42 (1.05)	4.56 (1.14)	4.59 (1.23)	0.04
HDL-cholesterol (mmol/L)^b^	1.37 (0.37)	1.26 (0.30)	1.24 (0.27)	0.002	1.39 (0.39)	1.39 (0.41)	1.32 (0.34)	NS
LDL-cholesterol (mmol/L)^b^	2.43 (0.80)	2.49 (0.66)	2.65 (0.76)	NS	2.57 (0.85)	2.69 (0.87)	2.65 (0.86)	NS
Non-HDL-cholesterol (mmol/L)^b^	2.84 (0.97)	2.90 (0.71)	3.08 (0.83)	NS	3.02 (0.99)	3.17 (1.10)	3.26 (1.31)	NS
Triglycerides (mmol/L)^c, d^	0.73 [0.52–1.05]	0.80 [0.58–1.04]	0.76 [0.57–1.24]	NS	0.85 [0.61–1.14]	0.86 [0.64–1.29]	0.97 [0.68–1.46]	0.003
Familial history of type 2 diabetes (%)	8.4	14.4	10.7	NS	14.3	22.7	24.1	<0.001*
Overt nephropathy (%)	0	0	0	NS	4.8	1.9	2.9	0.04
Coronary events (%)	0	0	0	NS	0.7	0.9	0.9	NS
Number of cardiovascular risk factors	0.90 (0.87)	0.95 (0.93)	1.01 (0.95)	NS	0.93 (0.87)	1.28 (0.84)	1.30 (0.78)	<0.001

Values are expressed in mean (SD), except where noted

*NS* not significant

* p for linear-by-linear association (Mantel–Haenszel test)

^a^ multiply by 18.018 to convert to mg/dL

^b^ multiply by 38.61 to convert to mg/dL

^c^ multiply by 88.5 to convert to mg/dL

^d^ median [interquartile range]

**Fig. 3:**
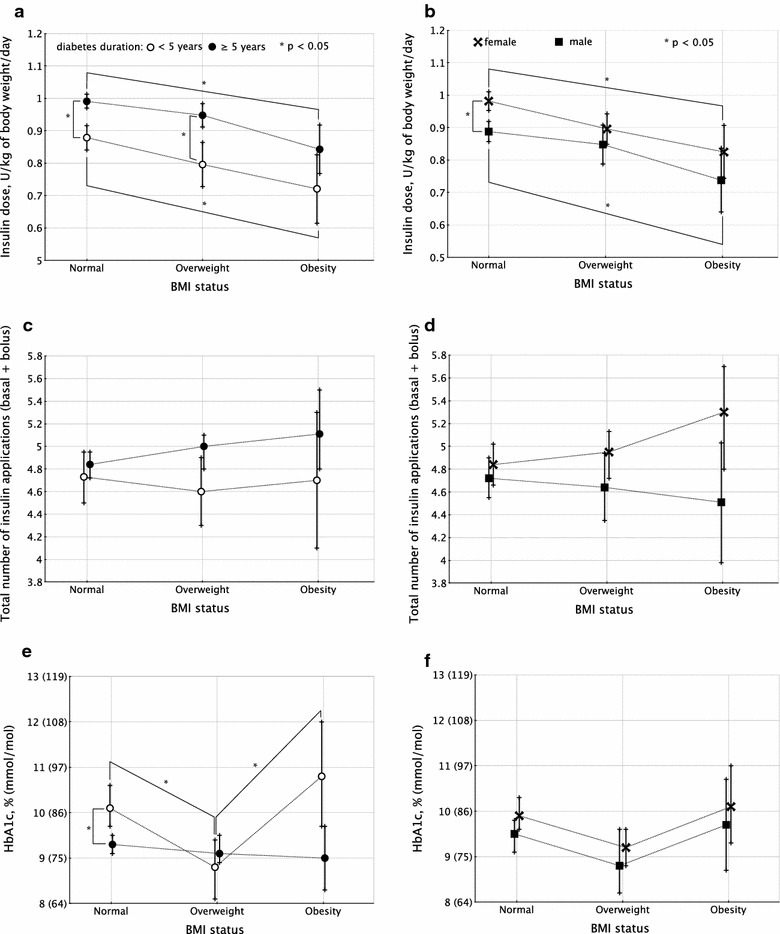
Estimated marginal means and 95 % CIs (according to BMI status) for insulin dose (**a** divided by diabetes duration; **b** divided by gender); total number of insulin applications (**c** divided by diabetes duration; **d** divided by gender); HbA1c (**e** divided by diabetes duration; **f** divided by gender). Significant differences (p < 0.05) are marked with *asterisks*

Insulin dose per body weight was significantly associated with age (partial-η^2^ 0.039), HbA1c (partial-η^2^ 0.054), HDL-cholesterol (partial-η^2^ 0.012), number of insulin applications (partial-η^2^ 0.073), BMI status (partial-η^2^ 0.008), and gender (partial-η^2^ <0.001). In the overall model, partial-η^2^ was 0.248, with p < 0.001. Estimated marginal means were lower in obese individuals than in those with normal BMI, in both genders and regardless of diabetes duration, as depicted on Fig. 3a, b (numeric values described in Additional file 1: Table S1).

Number of insulin applications was associated with HbA1c (partial-η^2^ 0.010), BSA (partial-η^2^ < 0.001), insulin dose per kg of body weight (partial-η^2^ 0.078), gender (partial-η^2^ 0.004). Partial-η^2^ for the overall model was 0.121 (p < 0.001). Estimated marginal means were similar, regardless of gender, BMI status and diabetes duration (depicted on Fig. 3c, d and Additional file 1: Table S1).

HbA1c was associated with age (partial-η^2^ 0.012), HDL-cholesterol (partial-η^2^ 0.010), non-HDL-cholesterol (partial-η^2^ 0.053), mean blood pressure (partial-η^2^ 0.005), body surface area (partial-η^2^ 0.007), insulin dose (U/kg of body weight/day) (partial-η^2^ 0.053), number of insulin applications (partial-η^2^ 0.009), BMI (partial-η^2^ 0.008), and familial history of type 2 diabetes (partial-η^2^ 0.005). Partial-η^2^ for the overall model was 0.163 (p < 0.001, power 100 %). Significant interaction was seen between diabetes duration and familial history of type 2 diabetes (partial-η^2^ 0.006, p = 0.005, power 84.3 %), and between BMI and diabetes duration (partial-η^2^ 0.007, p = 0.002, power 88.7 %). Estimated marginal means are depicted on Fig. 3e, f and Additional file 1: Table S1.

**Fig. 4:**
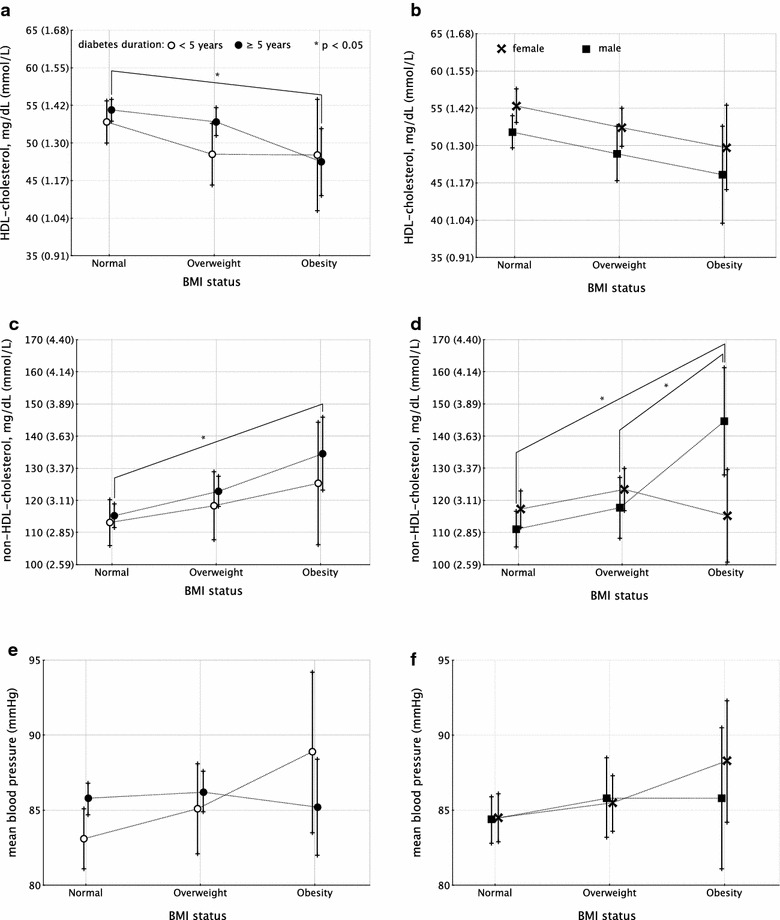
Estimated marginal means and 95 % CIs (according to BMI status) for HDL-cholesterol (**a** divided by diabetes duration; **b** divided by gender); non-HDL-cholesterol (**c** divided by diabetes duration; **d** divided by gender); mean blood pressure (**e** divided by diabetes duration; **f** divided by gender). Significant differences (p < 0.05) are marked with *asterisks*

HDL-cholesterol was associated with age (partial-η^2^ 0.009), non-HDL-cholesterol (partial-η^2^ 0.008), insulin dose per body weight (partial-η^2^ 0.013), and HbA1c (partial-η^2^ 0.010). Partial-η^2^ for the overall model was 0.079 (p < 0.001). Estimated marginal means are depicted on Fig. 4a, b and Additional file 1: Table S1.

Non-HDL-cholesterol was associated with age (partial-η^2^ 0.007, body surface (partial-η^2^ 0.005), HbA1c (partial-η^2^ 0.051), HDL (partial-η^2^ 0.009). Partial-η^2^ was 0.121 for the overall model (p < 0.001, power 100 %). Significant interaction was seen between gender and familial history of type 2 diabetes (partial-η^2^ 0.005, p = 0.002, power 85.9 %). Estimated marginal means are depicted on Fig. 4c, d and Additional file 1: Table S1.

Mean blood pressure showed partial-η^2^ 0.304 for the overall model (p < 0.001). Age (partial-η^2^ 0.057), BSA (partial-η^2^ 0.047), and HbA1c (partial-η^2^ 0.005) were associated with MBP. Estimated marginal means are depicted on Fig. 4e, f and Additional file 1: Table S1.

## Discussion

In this paper, we have studied a large sample of individuals with type 1 diabetes. About one third of this real-world sample was overweight/obese. Diabetes duration and intensity of insulin treatment were not related to weight excess. Insulin dose was inversely related to BMI status.

In univariate analysis, individuals with less than 5 years of diabetes duration were more obese, younger, and had lower HDL when compared to individuals with more than 5 years of diabetes duration. This finding could possibly be related to higher beta-cell residual function, since 5 years of diabetes duration could be regarded as a maximum time limit for its presence in type 1 diabetes [15]. Although the findings of obesity and lower HDL could be related to genetic predisposition for IR, this group had a lower frequency of type 2 diabetes in family history. This finding is compatible with the lower age range in this group, since there might be not enough time for older generations in the families to manifest type 2 diabetes.

In the group with higher diabetes duration, obese individuals had lower insulin doses per body weight. Nephropathy could influence insulin doses by decreasing its renal excretion. On the other hand, IR wouldn’t be higher in lean individuals (who had higher insulin doses) and our composite endpoint of nephropathy comprehends many individuals with mild or no renal disfunction. This lower insulin dose can be explained also by clinical inertia or fear of hypoglycaemia by both attending physicians and patients, since in this group HbA1c levels are far from recommended goals, although heterogeneity in the progression of beta-cell failure cannot be excluded given the findings of multivariable analysis. Longitudinal studies have previously demonstrated that C-peptide levels are higher at onset of type 1 diabetes in individuals with higher weight, although in a short term follow up of recently diagnosed patients [16].

Overweight and obesity were present in 31 % of our sample. Data about overweight and type 1 diabetes are very heterogeneous in literature, partly owing to different clinical criteria by which this clinical outcome is assessed. In a sample from Colorado, 16 % of youths with type 1 diabetes had BMIs above the 85th percentile for age (i.e., equivalent to both overweight and obese groups in our sample) upon diagnosis [17]. In 115 Spanish individuals with type 1 diabetes on intensive therapy, with a mean age of 12 years old and a mean diabetes duration of 5 years, about 30 % were overweight and 20 % were obese [18]. In a sample of American youths from 0 to 17 years old with diabetes, 16.3 % were obese and were classified as obese-indeterminate diabetes. They had significantly older age of onset and frequency of hypertension than type 1 diabetes [19].

Data for adults are not as widely available, and frequently analyzed jointly with data about children and adolescents. Both adolescents (mean age 15 years old) and adults (mean age 38 years old) gained weight after 1 year of follow up in the DCCT [20]. In the completed trial, after 6.5 years of follow up, there was approximately 13 % of adolescents in a sample with mean age around 27 years old [14]. In the Wisconsin Epidemiologic Study of Diabetic Retinopathy (WESDR), in a group of patients diagnosed before age 30 and followed for 4 years, weight variation went from losing 0.6 to gaining 3.4 kg in different quartiles [13]. Besides age range, recruitment criteria highly influence these numbers. In DCCT, individuals with more than 130 % of ideal body weight were excluded, making obesity upon diagnosis virtually non-existent in this sample and consequently in all follow-up studies [6].

As expected, insulin doses were higher in the group with longer disease duration, a finding compatible with the natural history of progressive insulin deficiency. Nevertheless, they were lower in obese than in normal weight individuals. Age could be a source of bias, since groups divided by diabetes duration had different ages and therefore different ages at diagnosis. Age at diagnosis is known to influence progression of beta-cell failure [21], with individuals diagnosed after 18 years old having higher baseline residual beta-cell function and less pronounced decay after several years of follow-up, when compared to individuals diagnosed in childhood. Since multivariable models have been corrected for body surface and age, this source of bias can be circumvented. Moreover, body surface was not significantly associated with insulin dose. Data from a Spanish group of individuals with type 1 diabetes and aged on average 17 years old showed that insulin dose per body surface but not with dose per kg of body weight was higher in individuals with metabolic syndrome. No combined analysis of both parameters has been performed, though. Insulin dose was moderately correlated with HbA1c and BMI [22]. In the initial pilot of the DCCT, insulin dose per body weight was higher in adolescents (0.94 U/kg) than in adults (0.65 U/kg), suggesting puberty-associated IR or clinical heterogeneity of late-onset type 1 diabetes rather than progression of beta-cell failure as an explanation for this variability [6]. Another possible hypothesis is that obesity could have different effects on insulin sensitivity in type 1 and type 2 diabetes.

Interestingly, intensity of insulin treatment, as measured by total number of insulin applications, showed no difference either among different BMI strata or diabetes duration subgroups. In spite of our cross-sectional design, this finding strongly suggests weight excess to be unrelated to intensity of insulin treatment. Moreover, owing to marked social and economic differences observed in our country which have been previously described [23], this sample has a particularly high number of individuals on conventional therapy (i.e., one or two insulin injections per day). This feature is especially suitable to assess the role of intense insulin therapy in this sample.

HbA1c didn’t show a clear trend of association with diabetes duration. This could be due to clinical heterogeneity or limitations of the cross-sectional design, since HbA1c is highly variable during follow-up, thus a single value could be misleading in this setting. The large variability in HbA1c levels could also be explained by factors related to residual pancreatic function and glucose/lipotoxicity, which were not directly evaluated in this study. There was a significant interaction between diabetes duration and familial history of type 2 diabetes, though, which could be related to clinical heterogeneity, partly explaining the irregular pattern of association between HbA1c and other variables. In literature, HbA1c was not correlated with BMI status in children and adolescents [18]. It showed no solid correlation with coronary artery disease (CAD), either [24].

Non-HDL-cholesterol was associated with weight excess in the predicted way. Obese individuals had higher non-HDL cholesterol than lean patients. Besides, interaction between gender and familial history of type 2 diabetes suggests this relationship is probably due to hereditary traits related to IR. Weight gain has been hypothesised to trigger genetic factors related to IR [25]. In this aspect, our sample is different from literature in exhibiting worse HDL in obese individuals. In the DCCT, all metabolic parameters worsened following weight gain, except for HDL, which remained stable [4]. This difference could point to a contribution of individuals obese upon diagnosis for our results, since these have not been excluded from our series. Interestingly, there was no difference in obesity, lipids, and familial history of type 2 diabetes in a substudy of DCCT/EDIC, when comparing individuals with negative and positive islet antibodies [25]. Other studies have also assessed the difficulty of utilising traditional clinical criteria to differentiate between type 2 diabetes and obese type 1 diabetes, with diabetic ketoacidosis being seen in 62 % of type 1 diabetes and 40 % of type 2. Despite the significant statistical difference, accuracy is extremely low [19].

Obese Spanish children and adolescents with type 1 diabetes had lower HDL and higher LDL than lean ones [18]. When assessing association of dyslipidaemia and CAD, individuals with type 1 diabetes and CAD had lower HDL and higher total cholesterol-to-HDL ratio than those without CAD [24]. Type 1 diabetes is classically associated with high HDL levels [2]. Nevertheless, obesity apparently is able to diminish this advantage [5]. Regarding risk of cardiovascular end points, the number of recorded CV events is too small to reach any valid conclusions in our sample. Besides, this analysis is beyond the scope of this study.

Blood pressure did not show a significant correlation with BMI in our sample. Hypertension has been previously shown to be more frequent in youths with type 2 and obese with indeterminate diabetes type than in type 1 diabetes [19]. Blood pressure is higher in individuals with type 1 diabetes with nephropathy or CAD than on complication-free subjects [24]. The presence of individuals in all age ranges with a low frequency of CAD and clinical nephropathy in our sample could be a possible explanation for these differences.

Given the study design, data must be further investigate in a prospective manner in order to confirm causal relationship among studied variables. External validity of the data must also be confirmed by studies from other populations, particularly in the adult age range, for which medical literature is still incipient in double diabetes.

Some limitations of the study should be addressed. The most important is the cross-sectional design. No causal relationship can be established with our data. Nevertheless, we feel the large sample and the fact patients have been unselected regarding BMI at diagnosis give a more realistic perspective of double diabetes in the heterogeneous scenario of type 1 diabetes. Another limitation is the absence of pancreatic autoantibodies in the diagnosis of type 1 diabetes. Although they at first could potentially contribute to differential diagnosis between type 1 diabetes and other subtypes of diabetes, there was no difference between lean and obese subjects regarding islet antibodies in the DCCT-EDIC [25]. Besides, even utilising this diagnostic tool, differential diagnosis can be difficult in a significant proportion of patients, as demonstrated in Finnish individuals [9, 10]. Moreover, although we hypothesised that clinical variability of residual beta-cell function as an explanation for the lower insulin dose in obese than in lean individuals, no direct measurements of C-peptide or other pancreatic function estimate were available in our sample. There is some evidence in literature showing residual beta-cell function to be higher in DD than in classical type 1 diabetes, though [26]. Furthermore, as seen by HbA1c levels far from the recommended goals, we can infer insulin treatment was not fully optimised in this sample. Nevertheless, multivariable models have been corrected for HbA1c, potentially tapering down the effects of metabolic decompensation on our main finding.

## Conclusions

In summary, approximately 30 % of individuals with type 1 diabetes are overweight in our population. The relationship between overweight and cardiovascular risk factors is more evident in type 1 diabetes with disease duration greater than 5 years. These data suggest a role for metabolic memory on this relationship also in type 1 diabetes, analogously to what is already known for type 2 diabetes. However, low HDL seems to be related to early obesity in type 1 diabetes and the metabolic environment associated to this condition. Weight excess was not specifically associated with intensity of insulin treatment and diabetes duration in this large group of individuals with type 1 diabetes. Obese individuals used lower insulin doses than lean ones, suggesting either suboptimal insulin treatment or clinical heterogeneity of type 1 diabetes, in which higher weight is possibly associated to higher residual beta-cell function. We can suggest that obesity associated to type 1 diabetes is multifactorial and occurs either upon diagnosis or in the first 5 years of disease. It is related to IR traits and occurs after this time period as a result of interaction between genetic background and hyperglycaemic milieu. However, both conditions represent subgroups of type 1 diabetic individuals that will probably need an early follow-up and management of cardiovascular risk factors. Concluding, these data suggest a broad landscape of clinical phenomena in the pathophysiology of DD, rather than simple progression of a homogeneous clinical entity.

## Additional file

10.1186/s13098-016-0143-7 Estimated marginal means and 95 % CIs of insulin dose, number of insulin applications, A1C, HDL-cholesterol, non-HDL-cholesterol, and mean blood pressure, in normal, overweight, and obese individuals according to gender and diabetes duration (numeric data corresponding to Figs. 3 and 4).

## Abbreviations

AER: albumin excretion rate
ANCOVA: analysis of covariance
BMI: body mass index
CAD: coronary artery disease
CI: confidence interval
CVRF: cardiovascular risk factor
DBP: diastolic blood pressure
DCCT: Diabetes Control and Complications Trial
DD: double diabetes
EDIC: Epidemiology of Diabetes Interventions and Complications
FPG: fasting plasma glucose
HbA1c: haemoglobin A1c
HDL: high-density lipoprotein
IR: insulin resistance
LDL: low-density lipoprotein
MBP: mean blood pressure
SBP: systolic blood pressure
UKPDS: United Kingdom Prospective Diabetes Study

## References

[1] Pozzilli P, Guglielmi C, Caprio S, Buzzetti R (2011). Obesity, autoimmunity, and double diabetes in youth. Diabetes Care.

[2] Cleland SJ, Fisher BM, Colhoun HM, Sattar N, Petrie JR (2013). Insulin resistance in type 1 diabetes: what is “double diabetes” and what are the risks?. Diabetologia.

[3] Teupe B, Bergis K (1991). Epidemiological evidence for “double diabetes”. Lancet.

[4] Purnell JQ, Hokanson JE, Marcovina SM, Steffes MW, Cleary PA, Brunzell JD (1998). Effect of excessive weight gain with intensive therapy of type 1 diabetes on lipid levels and blood pressure: results from the DCCT. Diabetes Control and Complications Trial. JAMA.

[5] Giuffrida FMA, Guedes AD, Rocco ER, Mory DB, Dualib P, Matos OS, Chaves-Fonseca RM, Cobas RA, Negrato CA, Gomes MB, Dib SA (2012). Brazilian Type 1 Diabetes Study Group (BrazDiab1SG). Heterogeneous behavior of lipids according to HbA1c levels undermines the plausibility of metabolic syndrome in type 1 diabetes: data from a nationwide multicenter survey. Cardiovasc Diabetol.

[6] The DCCT Research Group (1987). Diabetes Control and Complications Trial (DCCT): results of feasibility study. Diabetes Care.

[7] Cleland SJ (2012). Cardiovascular risk in double diabetes mellitus—when two worlds collide. Nat Rev Endocrinol..

[8] United Kingdom Prospective Diabetes Study (UKPDS) (1995). 13: Relative efficacy of randomly allocated diet, sulphonylurea, insulin, or metformin in patients with newly diagnosed non-insulin dependent diabetes followed for three years. BMJ.

[9] Lammi N, Taskinen O, Moltchanova E, Notkola I-L, Eriksson JG, Tuomilehto J, Karvonen M (2007). A high incidence of type 1 diabetes and an alarming increase in the incidence of type 2 diabetes among young adults in Finland between 1992 and 1996. Diabetologia.

[10] Lammi N, Blomstedt PA, Moltchanova E, Eriksson JG, Tuomilehto J, Karvonen M (2008). Marked temporal increase in the incidence of type 1 and type 2 diabetes among young adults in Finland. Diabetologia.

[11] Gale EAM (2006). Declassifying diabetes. Diabetologia.

[12] Gomes MB, Coral M, Cobas RA, Dib SA, Canani LH, Nery M, de Freitas MCF, Faria M, Felício JS, da Silva SC, Pedrosa H, Costa E, Forti A, Rea RR, Pires AC, Junior Montenegro R, Oliveira JE, Rassi N, Negrato CA (2012). Prevalence of adults with type 1 diabetes who meet the goals of care in daily clinical practice: a nationwide multicenter study in Brazil. Diabetes Res Clin Pract.

[13] Wing RR, Klein R, Moss SE (1990). Weight gain associated with improved glycemic control in population-based sample of subjects with type I diabetes. Diabetes Care.

[14] Adverse events and their association with treatment regimens in the diabetes control and complications trial. Diabetes Care. 1995;18(11):1415–27. 10.2337/diacare.18.11.1415 8722064

[15] The Diabetes Control and Complications Trial Research Group (1998). Effect of intensive therapy on residual beta-cell function in patients with type 1 diabetes in the diabetes control and complications trial. A randomized, controlled trial. Ann Intern Med.

[16] Cedillo M, Libman IM, Arena VC, Zhou L, Trucco M, Ize-Ludlow D, Pietropaolo M, Becker DJ (2015). Obesity, islet cell autoimmunity, and cardiovascular risk factors in youth at onset of type 1 autoimmune diabetes. J Clin Endocrinol Metab.

[17] Hummel K, McFann KK, Realsen J, Messer LH, Klingensmith GJ, Chase HP (2012). The increasing onset of type 1 diabetes in children. J Pediatr.

[18] Palomo Atance E, Giralt Muiña P, Ballester Herrera MJ, Ruiz Cano R, León Martín A, Giralt Muiña J (2013). [Prevalence of obesity and cardiovascular risk factors in a group of paediatric patients with type 1 diabetes]. An Pediatr (Barc).

[19] Lipton RB, Drum M, Burnet D, Rich B, Cooper A, Baumann E, Hagopian W (2005). Obesity at the onset of diabetes in an ethnically diverse population of children: what does it mean for epidemiologists and clinicians?. Pediatrics.

[20] The DCCT Research Group (1988). Weight gain associated with intensive therapy in the diabetes control and complications trial. Diabetes Care.

[21] Barker A, Lauria A, Schloot N, Hosszufalusi N, Ludvigsson J, Mathieu C, Mauricio D, Nordwall M, Van der Schueren B, Mandrup-Poulsen T, Scherbaum W, Weets I, Gorus F, Wareham N, Leslie R, Pozzilli P (2014). Age-dependent decline of β cell function in type 1 diabetes after diagnosis: a multi-centre longitudinal study. Diabetes Obes Metab.

[22] Valerio G, Iafusco D, Zucchini S, Maffeis C (2012). Study-Group on Diabetes of Italian Society of Pediatric Endocrinology and Diabetology (ISPED). Abdominal adiposity and cardiovascular risk factors in adolescents with type 1 diabetes. Diabetes Res Clin Pract.

[23] Gomes MB, Cobas RA, Matheus AS, Tannus LR, Negrato CA, Rodacki M, Braga N, Cordeiro MM, Luescher JL, Berardo RS, Nery M, Arruda-Marques MDCA, Calliari LE, Noronha RM, Manna TD, Zajdenverg L, Salvodelli R, Penha FG, Foss MC, Foss-Freitas MC, Pires AC, Robles FC, Guedes M, Dib SA, Dualib P, Silva SC, Sepulvida J, Almeida HG, Sampaio E, Rea R, Faria ACR, Tschiedel B, Lavigne S, Cardozo GA, Azevedo MJ, Canani LH, Zucatti AT, Coral MHC, Pereira DA, Araujo LA, Tolentino M, Pedrosa HC, Prado FA, Rassi N, Araujo LB, Fonseca RMC, Guedes AD, Matos OS, Faria M, Azulay R, Forti AC, Façanha C, Montenegro AP, Montenegro R, Melo NH, Rezende KF, Ramos A, Felicio JS, Santos FM, Jezini DL (2012). Regional differences in clinical care among patients with type 1 diabetes in Brazil: Brazilian Type 1 Diabetes Study Group. Diabetol Metab Syndr.

[24] Zgibor JC, Piatt GA, Ruppert K, Orchard TJ, Roberts MS (2006). Deficiencies of cardiovascular risk prediction models for type 1 diabetes. Diabetes Care.

[25] Purnell JQ, Dev RK, Steffes MW, Cleary PA, Palmer JP, Hirsch IB, Hokanson JE, Brunzell JD (2003). Relationship of family history of type 2 diabetes, hypoglycemia, and autoantibodies to weight gain and lipids with intensive and conventional therapy in the diabetes control and complications trial. Diabetes.

[26] Yu HW, Lee YJ, Cho WI, Lee YA, Shin CH, Yang SW (2015). Preserved C-peptide levels in overweight or obese compared with underweight children upon diagnosis of type 1 diabetes mellitus. Ann Pediatr Endocrinol Metab.

