# Supplementary material for: Double-diabetes in a real-world sample of 2711 individuals: associated with insulin treatment or part of the heterogeneity of type 1 diabetes?
Source: Diabetol Metab Syndr. 2016 Mar 22;8:28. doi: 10.1186/s13098-016-0143-7 (PMC4804538; doi:10.1186/s13098-016-0143-7)
Supplement: Supplementary file 1 — 10.1186/s13098-016-0143-7 Estimated marginal means and 95 % CIs of insulin dose, number of insulin applications, A1C, HDL-cholesterol, non-HDL-cholesterol, and mean blood pressure, in normal, overweight, and obese individuals according to gender and diabetes duration (numeric data corresponding to Figs. 3 and 4). [file 13098_2016_143_MOESM1_ESM.pdf]

Online Supplemental Table 1: Estimated marginal means and 95% CIs of insulin dose, number of insulin applications, A1C, HDL-cholesterol, non-HDL-cholesterol, and mean blood pressure, in normal, overweight, and obese individuals according to gender and diabetes duration (numeric data corresponding to Figures 3 and 4).

		**Normal**	**Overweight**	**Obesity**
**Insulin dose per body weight (U/kg)**	**Males**	0.888 (0.857-0.919)	0.848 (0.788-0.908)	0.738 (0.640-0.837)
	**Females**	0.982 (0.953-1.011)	0.896 (0.849-0.943)	0.825 (0.744-0.907)
	**Diabetes duration < 5 years**	0.879 (0.841-0.916)	0.796 (0.728-0.864)	0.721 (0.615-0.826)
	**Diabetes duration ≥ 5 years**	0.991 (0.970-1.013)	0.948 (0.912-0.984)	0.843 (0.768-0.918)
**Number of insulin applications**	**Males**	4.721 (4.547-4.896)	4.642 (4.347-4.938)	4.508 (3.984-5.032)
	**Females**	4.841 (4.663-5.018)	4.925 (4.720-5.131)	5.293 (4.841-5.745)
	**Diabetes duration < 5 years**	4.727 (4.503-4.950)	4.595 (4.263-4.927)	4.687 (4.091-5.283)
	**Diabetes duration ≥ 5 years**	4.835 (4.721-4.950)	4.973 (4.825-5.120)	5.115 (4.758-5.471)
**A1C (%)**	**Males**	9.50 (9.15-9.82)	8.76 (8.19-9.32)	9.74 (8.73-10.74)
	**Females**	9.97 (9.63-10.31)	9.19 (8.80-9.69)	10.13 (9.27-11.00)
	**Diabetes duration < 5 years**	10.15 (9.72-10.58)	8.79 (8.15-9.43)	10.85 (9.71-12.0)
	**Diabetes duration ≥ 5 years**	9.31 (9.09-9.53)	9.16 (8.88-9.45)	9.02 (8.33-9.70)
**HDL-cholesterol (mg/dL)**	**Males**	51.9 (49.7-54.0)	48.9 (45.3-52.6)	46.2 (39.6-52.6)
	**Females**	55.4 (53.1-57.6)	52.4 (49.9-55.0)	49.8 (44.1-55.4)
	**Diabetes duration < 5 years**	52.8 (50.0-55.6)	48.5 (44.4-52.6)	48.4 (41.0-55.8)
	**Diabetes duration ≥ 5 years**	54.4 (53.0-55.8)	52.8 (51.0-54.7)	47.5 (43.0-51.9)
**non-HDL-cholesterol (mg/dL)**	**Males**	111.0 (105.5-116.6)	117.7 (108.2-127.1)	144.6 (127.9-161.3)
	**Females**	117.2 (111.5-122.9)	123.4 (116.8-129.9)	115.2 (100.7-129.6)
	**Diabetes duration < 5 years**	113.1 (105.9-120.2)	118.3 (107.7-128.9)	125.3 (106.2-144.3)
	**Diabetes duration ≥ 5 years**	115.2 (111.6-118.9)	122.8 (118.1-127.5)	134.5 (123.2-145.9)
**Mean blood pressure (mmHg)**	**Males**	84.4 (82.8-85.9)	85.8 (83.2-88.5)	85.8 (81.1-90.5)
	**Females**	84.5 (82.9-86.1)	85.5 (83.6-87.3)	88.3 (84.2-92.3)
	**Diabetes duration < 5 years**	83.1 (81.1-85.1)	85.1 (82.1-88.1)	88.9 (83.5-94.2)
	**Diabetes duration ≥ 5 years**	85.8 (84.7-86.8)	86.2 (84.9-87.6)	85.2 (82.0-88.4)
